# No difference in clinical outcome between quadriceps tendon anterior cruciate ligament reconstruction with and without bone block: Results from the Danish Knee Ligament Registry

**DOI:** 10.1002/ksa.12451

**Published:** 2024-09-20

**Authors:** Martin Lind, Torsten Nielsen

**Affiliations:** ^1^ Department of Orthopaedics Aarhus University Hospital Aarhus Denmark

**Keywords:** ACL reconstruction, clinical outcomes, hamstring tendon, patellar tendon, quadriceps tendon

## Abstract

**Purpose:**

The quadriceps tendon (QT) has recently gained increasing interest as an anterior cruciate ligament reconstruction (ACLR) graft due to minimally invasive harvesting techniques and low donor site morbidity. QT grafts can be used both with a patella bone block and as complete soft tissue grafts. However, it is unknown whether the QT graft type affects clinical outcomes. This study used data from the Danish Knee Ligament Reconstruction Registry (DKRR) to compare revision rates, knee stability and subjective clinical outcomes in patients who underwent ACLR with QT graft with bone block (QT‐B) or soft tissue only (QT‐S).

**Methods:**

Patients who underwent primary ACL reconstruction with QT autografts documented in the DKRR were included and divided into the QT‐B (*n* = 925) and QT‐S (*n* = 659) groups. The clinical outcome was evaluated using objective‐instrumented knee stability, pivot shift test, knee injury osteoarthritis outcome score (KOOS) and Tegner activity scores for the two cohorts performed at the 1‐year follow‐up. The overall revision rates were determined as well.

**Results:**

Revision rates at 2 years were equally low in both graft groups at 2.8%. Similarly, post‐operative knee laxity was equal at 1.5 (1.4) and 1.6 (1.4) mm side‐to‐side laxity, respectively. However, QT‐B exhibited a reduced post‐operative positive pivot shift of 22% compared with 31% for QT‐S. Although the subjective outcomes were equal for the KOOS and Tegner activity scale scores at the 1‐year follow‐up, reduced improvements in KOOS were observed for QT‐B compared to QT‐S.

**Conclusion:**

ACL with a QT autograft harvested either with a bone block or as a soft tissue graft exhibited comparable revision rates and sagittal knee stability. Furthermore, ACL reconstruction using a QT graft with a bone block achieved better rotational stability with less pivot shift than ACL reconstruction using complete soft tissue QT grafts.

**Level of Evidence:**

Level III.

**Abbreviations**
ACL: anterior cruciate ligament
ACLR: anterior cruciate ligament reconstruction
DKRR: Danish Knee Ligament Reconstruction Registry
KOOS: knee osteoarthritis and injury outcome score
QT: quadriceps tendon
QT‐B: quadriceps tendon graft with bone block
QT‐S: quadriceps tendon graft with soft tissue

## INTRODUCTION

The quadriceps tendon (QT) has recently gained increasing interest as anterior cruciate ligament reconstruction (ACLR) graft due to the introduction of minimal invasive harvesting techniques and low donor site morbidity [4, 25, 26].

Quadriceps tendon ACLR can be performed using two types of graft‐harvesting techniques. The first is a pure soft tissue graft (QT‐S) harvest technique that uses a central and 7–8 cm long partial or full thickness part of the tendon. The second involves a combined soft tissue and proximal patellar bone block graft harvest with a 7–8 cm long part of the tendon, together with a 20 mm bone block from the quadriceps tendon insertion area at the proximal patella (QT‐B).

Regarding the impact of these two quadriceps (QT) graft types on clinical outcomes, a graft with a bone block could potentially have improved healing in bone tunnels owing to bone‐to‐bone healing. Furthermore, a longer overall graft enables better fixation and tunnel healing of the soft tissues of the graft. These theoretical benefits of the QT‐B graft may result in better post‐operative knee laxity and lower graft failure compared to the QT‐S due to bone‐to‐bone healing. However, QT‐B harvesting is associated with a higher risk of patellar fractures than QT‐S harvesting [6, 9]. Moreover, a potential benefit of QT‐S is that it can be used in cases with open physes.

A recent systematic review by Meena et al. investigated the outcomes of QT ACLR with and without a bone block [17] and found that both grafts are safe and viable options for ACL, with comparable clinical outcomes, complications, and revision rates. Another recent review by Crum et al. found that QT‐B was associated with higher levels of complications and increased rotatory instability, although graft failure and subjective outcomes were similar for both graft types [3].

However, no studies included in these systematic reviews directly compared the outcomes of the two QT graft techniques. Among them, only one registry study comparing QT, hamstring, and patellar tendon grafts compared the revision rates between the two QT graft techniques. A subgroup analysis of 531 QT grafts with and without bone block showed no difference in revision rates, although the exact data were not presented [15]. Furthermore, several studies have found that QT grafts for ACLR produce good clinical outcomes with low donor site morbidity, good subjective outcomes, good knee stability, and revision/failure rates comparable to those of other major graft types, such as hamstring and patellar tendon grafts [2, 5, 7, 11, 14, 16, 18, 22].

The Danish Knee Ligament Reconstruction Registry (DKRR) [13] now contains the details of more than 1500 QT ACLRs registered from 2012 to 2023, thus permitting a comparison of the revision rates and objective clinical outcomes of QT grafts with and without a bone block.

The purpose of the present study was to examine data obtained from the DKRR to compare the revision rates, knee stability and subjective clinical outcomes of patients who underwent ACLR with QT graft with and without a bone block. It was hypothesized that ACLR with QT‐S would result in more knee laxity and higher revision rates than ACLR with QT‐B, owing to the potential inferior soft tissue graft for bone tunnel healing.

## MATERIALS AND METHODS

This study was approved by the Regional Center for Clinical Quality Development and the National Data Protection Agency (approval number 1‐16‐02‐65‐17). In Denmark, no written consent is necessary to conduct studies based on data from the National Board of Health‐approved national healthcare registries.

This study was conducted using data from the DKRR, a prospective nationwide web‐based clinical database initiated in 2005. This registry contains data on both primary and revision anterior and posterior cruciate ligament reconstructions, as well as collateral ligament and multi‐ligament reconstructions performed in Denmark. Both public and private hospitals supply data to the registry [12]. The operating surgeon recorded pre‐operative, operative, and 1‐year follow‐up data using a standardized form on a secure Internet portal. Furthermore, patients independently reported subjective knee function using self‐assessed instruments and the knee injury osteoarthritis outcome score (KOOS) and Tegner activity scale scores [21, 24]. The surgeon or physician assistant recorded objective‐instrumented Lachman laxity and pivot shift test outcome at the 1‐year follow‐up. The patients entered their KOOS and Tegner activity scale data into a web‐based form before surgery and 1 year after surgery.

### Patients

In Denmark, QT graft usage began to gain popularity in 2012. Therefore, the patient cohort in this study was limited to those who underwent surgery between 2014 and 2021. The inclusion criterion was primary ACL using a QT autograft. A total of 1584 ACL reconstructions were eligible for inclusion. The exclusion criteria were previous ligament procedure, age <16 years, previous contralateral ACL injury, other graft types, and any previous meniscus or cartilage surgery on the affected knee. Two study populations were identified based on the QT graft type for ACL reconstruction: patients with QT autografts, including bone blocks (QT‐B) (*n* = 925), and patients with QT autografts without bone blocks (QT‐S) (*n* = 659).

The completeness of surgical registration was determined by correlating the registry data with patient data from the National Registry of Patients, in which all public and private hospital contacts and procedures were registered. The overall completeness of ACL procedure registration in the ACL registry was 91% for the data in the present study [20]. The completeness of the 1‐year follow‐up objective knee stability assessment was 53%. The completeness of patient‐reported outcome data was 34% pre‐operatively and 25% at the 1‐year follow‐up. Notably, a validation study conducted on the DKRR demonstrated no difference in epidemiologic characteristics, clinical outcomes, or revision rates between respondents and nonrespondents [20].

### Patient characteristics

The average age of the patients was 26.6 years (range, 16–70 years), and 62% of patients were male. Sports participation was the cause of injury in 85.5% of the cases. The only difference between the two QT graft groups was related to cartilage injury at the time of surgery where a minor difference of 2% was found (Table 1).

**Table 1:** Patient characteristics of the two graft groups.

Graft groups	QT‐B	QT‐S	*p* Value
*N* total	925	659	
Age, mean ± SD (range)	26.3 ± 9.0 (16–67)	27.1 ± 8.2 (16–57)	ns
Male sex, *n* (%)	556 (60.1)	426 (64.6)	ns
Injury in sports, *n* (%)	804 (86.9)	557 (84.5)	ns
Pre‐operative laxity (mm), mean ± SD	4.9 ± 1.8	4.8 ± 1.9	ns
Pre‐operative pos. pivot shift, *n* (%)	867 (94.4)	535 (92.4)	ns
Meniscus injury, *n* (%)	428 (46.3)	335 (50.8)	ns
Cartilage injury, *n* (%)	229 (24.8)	173 (26.2)	*p* = 0.03

Abbreviations: QT‐B, quadriceps tendon with bone block; QT‐S, quadriceps tendon with soft tissue only.

### Outcomes

The primary outcome examined in this study was ACLR failure, expressed as the need for revision ACLR, as decided by individual surgeons based on continued instability or reinjury.

The secondary outcome investigated was objective knee stability as estimated using instrumented sagittal knee stability testing and pivot‐shift tests. The sagittal stability test measures the difference in sagittal stability between the operated and healthy knees using the Knee Translation Instrument 1000 (KT‐1000) or the Rolimeter. The pivot shift test is a dynamic but passive test of the knee that measures the rotational and anterior tibial translation stability of the ACL. The pivot‐shift test was graded on a 4‐point scale, where 0 = *negative*, 1 = *glide*, 2 = *clunk* and 3 = *gross* [10]. Pivot‐shift data were classified into negative and positive pivot‐shift tests, with the latter including all levels of positive pivot‐shift tests.

### Statistical analyses

Descriptive data are presented as means and standard deviations and were compared using the Student's *t* test and the chi‐square test for proportional data. Furthermore, this study employed Cox regression analysis to compare the revision risk within the first 2 years after primary ACL surgery for patients in the two graft groups. By applying the Kaplan–Meier method, we estimated the revision probability of the two graft groups for the entire follow‐up period. Hazard ratios were computed as a measure of relative risk (RR), both crudely and after adjusting for potential confounding factors. The confounding factors included in the analysis were sex, age (≤20 and >20 years), cartilage damage >1 cm^2^ present (no/yes or missing data), and surgical treatment of meniscal injury either resection or repair (yes/no or missing data). These confounding factors were chosen based on known factors that influence ACL reconstruction outcomes. Statistical significance was set at *p* < 0.05. significant. All statistical analyses were performed using Stata Version 15 (StataCorp. 2023. Stata Statistical Software: Release 18. College Station, TX, StataCorp LLC) software.

## RESULTS

### Revision rates

**Figure 1:**
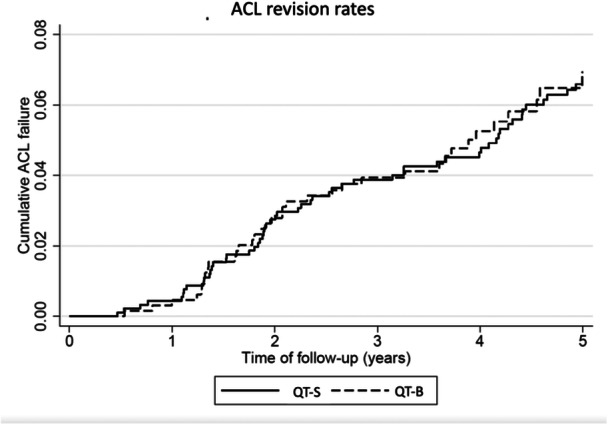
Kaplan–Meier curves showing ACL revision rates for the two quadriceps tendon graft types. ACL, anterior cruciate ligament; QT‐B, quadriceps tendon graft with bone block from the patella; QT‐S, quadriceps tendon graft with soft tissue only.

The 2‐year adjusted revision rates for QT‐B and QT‐S were 2.75% and 2.80%, respectively, with no significant differences in the adjusted hazard ratios. The Kaplan–Meier revision rates for the two QT graft types are shown in Figure 1. The overall revision rate, including 7 years of follow‐up, was calculated to be 8.0% (6.2–10.4) and 8.2% (5.7–11.6) for QT‐B and QT‐S, respectively.

### Objective knee laxity

Knee laxity, as determined using the knee arthrometer, was significantly decreased by ACLR surgery in both QT graft groups (Table 2). At the 1‐year follow‐up, sagittal instrumented laxity for QT‐B and QT‐S was 1.5 (1.4) mm and 1.6 (1.4) mm, respectively (ns).

**Table 2:** Post‐operative objective knee laxity and negative Pivot Shift results after ACL reconstruction. Knee laxity as measured using instrumented side‐to‐side difference laxity using the KT‐1000 device or the Rolimeter.

	QT‐B	QT‐S	*p* Value
*N*	*n* = 670	*n* = 439	
Post‐operative laxity (mm), mean ± SD	1.5 ± 1.4^a^	1.6 ± 1.4^a^	ns
*N*	*n* = 705	*n* = 328	
Post‐operative pos. pivot shift, *n* (%)	154 (21.8)^a^	147 (28.9)^a^	<0.001

Abbreviations: N, number of patients for the data parameter; QT‐B, quadriceps tendon with bone block; QT‐S, quadriceps tendon with soft tissue only.

a Significant reduction in laxity from the pre‐operative to post‐operative period.

However, a positive post‐operative pivot‐shift test was found for QT‐B and QT‐S in 21.8% and 28.9% of patients, respectively, with QT‐B grafts having significantly positive pivot‐shift than QT‐S grafts (*p* < 0.001) (Table 2).

### Subjective clinical outcomes

The KOOS demonstrated improvements for both QT graft cohorts in all five subscales from the pre‐operative to 1‐year follow‐up state (Table 3). When comparing the 1‐year post‐operative outcomes between the two graft types, the QT‐B ACLR group exhibited significantly lower improvements in the KOOS symptoms and KOOS Sports subscales than the QT‐S group (*p* = 0.01).

**Table 3:** Post‐operative subjective outcomes for the KOOS and Tegner activity scale.

	QT‐B	QT‐S	QT‐B	QT‐S	QT‐B	QT‐S
	Pre‐OP		Post‐OP		Difference	
KOOS Pain, mean (SD)	71.5 (6)	71.4 (17)	82.9 (14)^a^	84.5 (13)^a^	10.5 (16)	12.2 (16)
KOOS Symp, mean (SD)	72.2 (15)	70.0 (16)	75.8 (16)^a^	78.6 (15)^a^	3.6 (19)	7.9 (19)^b^
KOOS ADL, mean (SD)	80.3 (16)	79.2 (18)	89.4 (11)^a^	90.8 (11)^a^	8.8 (15)	9.9 (16)
KOOS Sport, mean (SD)	39.2 (25)	39.4 (26)	62.1 (24)^a^	66.6 (22)^a^	19.6 (27)	26.0 (27)^b^
KOOS QoL, mean (SD)	39.3 (16)	38.4 (16)	57.2 (20)^a^	57.0 (19)^a^	17.3 (21)	16.9 (20)
Tegner activity, median (range)	3 (2–5)	3 (2–5)	5 (2–10)	5 (2–10)	2	2

Abbreviations: Difference, difference in KOOS between pre‐operatively and 1‐year follow‐up; Pre‐OP, KOOS pre‐operatively; Post‐OP, KOOS at the 1‐year follow‐up; QT‐B, quadriceps tendon graft with bone block; QT‐S, quadriceps tendon graft with soft tissue only.

a Significant difference between the pre‐operative and post‐operative values.

b Significant difference between post‐operative QT‐B and QT‐S.

### Tegner activity scores

Tegner activity scale scores improved for both graft cohorts (*p* < 0.001), with pre‐operative scores of 3 (2–5) for both QT‐B and QT‐S, respectively, improving to 5 (2–10) for both graft groups, with no difference between graft groups at the 1‐year follow‐up (Table 3).

## DISCUSSION

The primary finding of this study was that ACL reconstructions using a QT autograft with either a bone block or a complete soft tissue graft demonstrated similar low revision rates and good sagittal knee stability. Therefore, the hypothesis that QT‐S ACLR results in greater knee laxity and higher failure rates could not be confirmed.

The low failure/revision rates of approximately 2.8% at 2 years follow‐up for both techniques correspond well with the results of two systematic reviews that found revision rates of 2.5% and 1.6% for QT‐B and QT‐S, respectively [3, 17]. Regarding sagittal laxity, the present study found instrumented side‐to‐side laxity to be around 1.5 mm for both groups, whereas the two systematic reviews observed laxities ranging from 0.5 to 2.1 mm for both techniques.

Regarding subjective knee function outcomes, the difference in improvements in KOOS symptoms and sports subscales between the pre‐operative and post‐operative stages at the 1‐year follow‐up, indicated less improvement for QT‐B than for QT‐S. The difference in improvement was 4.3 for KOOS symptoms and 7.4 for KOOS Sports. A potential explanation for these differences could be the extra symptoms related to donor site morbidity arising from bone block harvest during QT‐B ACLR. However, lower subjective scores for QT‐B than for QT‐S were not observed in a systematic review by Crum et al., where the IKDC score was generally lower for QT‐S than for QT‐B [3]. A review by Meena et al. found similar subjective outcomes for the two graft types [17]. Therefore, previous literature does not support poorer subjective outcomes with QT‐B ACLR, as suggested in the present study.

A secondary finding of this study was that ACL reconstruction with QT‐B achieves better rotational stability than ACL reconstruction using complete QT‐S grafts, as indicated by post‐operative positive pivot‐shift observed in only 22% of patients with QT‐B compared to 29% for QT‐S ACL reconstruction. A potential cause of better rotatory stability in QT‐B ACLR could be the stiffer graft construct obtained by bone‐to‐bone healing, which may have a more pronounced impact on the more complex and combined loading pattern of the pivot shift without affecting the more unidirectional sagittal knee stability measure. This finding contrasts with those of Crum et al., who found increased rotatory instability in patients operated with QT‐B compared to QT‐S, with 16% positive pivot shift compared to 0%, respectively. However, this finding for the QT‐S was based on a review of only three studies comprising a total of 116 patients.

Furthermore, QT‐B is associated with certain complications that are not found in QT‐S, such as patellar fractures, which are seen in approximately 1% of cases [3, 17]. This could be a valid argument in favour of choosing QT‐S over QT‐B. Although donor site morbidity for QT‐B ACLR is lower than that for hamstring and patellar tendons [14, 16], bone block harvest for QT‐B might still affect symptom levels at the 1‐year follow‐up as seen in the present study. Moreover, using a bone block might affect sports activities, as quadriceps muscle function in QT‐B ACLR remains impaired even at 1‐year follow‐up, as observed in a level 1 study investigating muscle function [23]. Muscle impairment can also affect sports functioning. However, no difference between the two QT techniques was identified in the Tegner activity scale score in the present study. In addition, as the observed differences in KOOS did not exceed the minimal clinically relevant difference, which was approximately 10–15 points for the different KOOS subscales, they might not be clinically relevant. Notably, in previous systematic reviews, the comparison of patient‐reported outcomes revealed no differences in the KOOS, IKDC, and Tegner scores [3, 17], which further supports the fact that there is no clinically relevant difference in the subjective outcomes of QT‐B and QT‐S ACLR.

A key concern pertaining to QT‐S has been insufficient graft incorporation, with typically only 1.5–2.0 cm of graft reaching into the femoral and tibial bone tunnels when harvesting a typically 7‐cm‐long QT‐S graft. The small amount of graft tissue in the bone tunnels usually necessitates the use of graft fixation techniques, which can lead to insufficient graft incorporation. In particular, suspensory graft fixation techniques have been associated with a higher risk of tunnel widening and graft laxity [8, 19]. This is supported by the findings of a recent biomechanical study by Arakgi et al., who found that for cyclic loading, QT‐S grafts using suspensory methods of fixation exhibit significantly greater displacement than QT‐B [1]. This could lead to a greater risk of graft laxity, and in turn, a higher likelihood of graft failure. However, neither the present study nor systematic reviews identified any issues related to laxity or failure rates, indicating that pure soft tissue‐based QT graft ACLR is safe and produces good clinical outcomes. Notably, the graft fixation benefit of QT‐B, in which the bone block ensures optimal healing due to bone‐to‐bone healing, did not appear to be relevant in the context of improved post‐operative stability or risk of failure.

Therefore, the clinical implications of the present comparative study confirm that the results of QT‐S and QT‐B ACLR show similarly good knee stability, as well as subjective and low failure outcomes. However, as QT‐S involves fewer patella‐related complications, it may be preferred over QT‐B for ACLR using QT grafts.

The most significant strength of this study is its large number of patients (>1500), which is crucial for accurate evaluation of the rare failure parameter of a revision reoperation, whose incidence rate typically remains below 5% at 2 years for ACL reconstructions [13]. Another strength was the inclusion of patients from several centres nationwide, which ultimately contributed to achieving a high completeness value of >90%. Moreover, the use of registry data provides more generalizable results because they represent a generalized surgical population.

However, this study had some limitations. Selection bias is an important issue when using registry data, especially in cases involving new techniques such as QT graft usage, because the motivation for using a new graft type is not recorded in a registry. Furthermore, the evaluation of knee stability outcomes using instrumented knee laxity measurements and pivot shift tests is usually conducted by surgeons in most clinics. This may lead to a bias towards better stability measurements, which should be considered when evaluating objective outcomes. In addition, the completeness of the knee stability outcome data was not optimal; therefore, these data should be interpreted with caution. Patient‐reported outcomes have low completeness, which necessitates careful evaluation of these parameters. Additionally, although revision surgery was used as the endpoint of failure in this study, this parameter did not account for patients with subjective or objective graft failure who did not undergo revision surgery.

## CONCLUSION

ACL reconstruction with QT autografts harvested either with a bone block or as a complete soft tissue graft exhibited comparable revision rates and sagittal knee stability. Furthermore, ACL reconstruction using a QT graft with a bone block yielded better rotational stability than ACL reconstruction using complete soft tissue QT grafts.

## AUTHOR CONTRIBUTIONS

**Martin Lind**: Project idea; data interpretation; manuscript preparation. **Torsten Nielsen**: Data analysis and interpretation; manuscript preparation.

## CONFLICT OF INTEREST STATEMENT

The authors declare no conflict of interest.

## ETHICS STATEMENT

This study was approved by the Regional Center for Clinical Quality Development and the National Data Protection Agency (approval number 1‐16‐02‐65‐17). In Denmark, no written consent is necessary to conduct studies based on data from the National Board of Health‐approved national healthcare registries. Patient consent is not needed for registry studies with data from Danish national clinical registries.

## Data Availability

No raw data are available since such data distribution is not allowed by the Danish Knee Ligament Reconstruction Registry.
